# A case of a drug reaction to sulfasalazine in a patient infected with HIV

**DOI:** 10.4102/sajhivmed.v19i1.829

**Published:** 2018-12-03

**Authors:** Leanne Swart, Elise Schapkaitz, Anima Baiden

**Affiliations:** 1 Department of Molecular Medicine and Haematology, National Health Laboratory Service, University of the Witwatersrand, South Africa; 2 Department of Haematology, National Health Laboratory Service, University of the Witwatersrand, South Africa

## Abstract

**Introduction:**

The diagnosis of drug reaction with eosinophilia and systemic symptoms (DRESS) in human immunodeficiency virus (HIV) patients on multiple drugs with concomitant disorders presents a diagnostic challenge.

**Patient presentation:**

We describe a case of a drug reaction to sulfasalazine in a 46 year old HIV-infected female with concurrent rheumatoid arthritis which presented atypically with a marked peripheral blood plasmacytosis mimicking a lymphoproliferative neoplasm.

**Management and outcome:**

A diagnosis of DRESS was made in conjunction with the laboratory and clinical presenting findings. Sulfasalazine was immediately discontinued. The mucocutaneous rash and systemic symptoms (which included fever, lymphadenopathy and multi-organ dysfunction) resolved with supportive treatment. This included topical and systemic corticosteroids.

**Conclusion:**

In conclusion, it is important to consider drug reactions when evaluating patients infected with HIV.

## Introduction

Drug reaction with eosinophilia and systemic symptoms (DRESS) is a severe adverse drug reaction. Drug hypersensitivity reactions are common in patients infected with human immunodeficiency virus (HIV), secondary to drugs prescribed for the treatment of HIV and for the prevention of opportunistic illnesses. The diagnosis of DRESS in HIV patients on multiple drugs with concomitant disorders presents a challenge. We present an unusual case of a drug reaction to sulfasalazine in a patient infected with HIV with concurrent rheumatoid arthritis (RA), which presented as a marked peripheral blood plasmacytosis.

## Case report

A 46-year-old female patient with RA, known to the Charlotte Maxeke Academic Hospital’s rheumatology outpatient department, presented with a one-week history of fever (> 38 °C) and a generalised skin rash requiring hospitalisation. She gave a history of starting sulfasalazine two weeks prior, for the management of RA. She had no known allergies. On past medical history, she was also HIV-positive with an absolute CD4+ count of 411 cells/µL and a lower than detectable viral load. Other chronic medications (for > 3 months) included hydroxychloroquine for RA, risperidone for psychiatric manifestations of HIV, and antiretroviral therapy, namely tenofovir, lopinavir with ritonavir, and lamivudine. On examination, she was found to have significant (> 1.5 cm) bilateral cervical and left submental lymphadenopathy associated with severe periorbital oedema. On skin and mucosal examination, her palms and soles were indurated, her lips showed superficial mucosal erosions and there were widespread urticarial papules and target lesions on her face, trunk and extremities. A differential diagnosis including erythema multiforme major, vasculitis and acute drug eruptions such as Steven-Johnson Syndrome and toxic epidermal necrolysis were considered.

**FIGURE 1:**
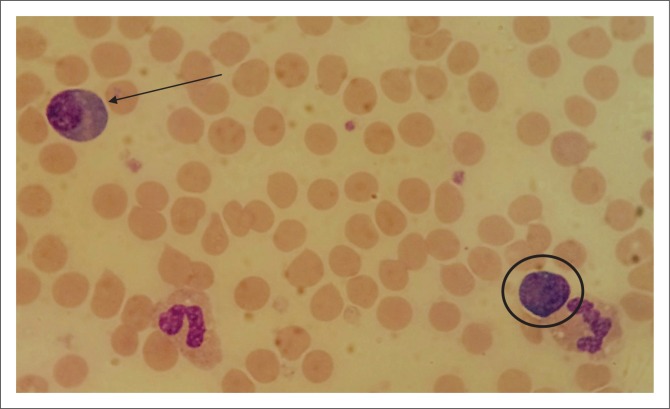
Giemsa-stained peripheral blood smear at ×100 magnification showing plasmacytoid (arrow) and atypical lymphocytes (circle). Lymphocytes ranged from small to intermediate in size with deeply basophilic cytoplasm, eccentric nuclei and nuclear folding.

**FIGURE 2:**
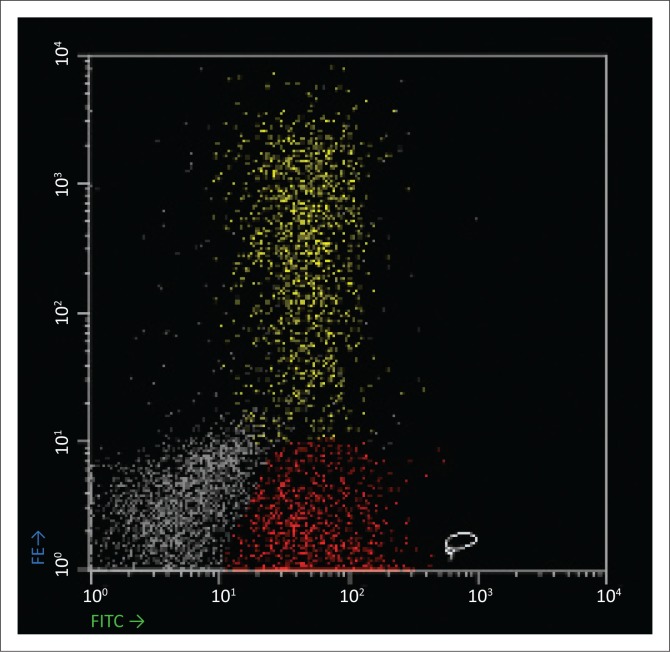
Immunophenotypic analysis of the peripheral blood performed on a dual laser FACSCalibur equipped with CellQuest Pro and PAINT-A-Gate Pro software. CD19 is the monoclonal antibody on FITC and CD138 is the monoclonal antibody on PE. There is a population of ~20% – 25% plasma cells (yellow) which express a range of CD138 and CD19, and a population of ~20% B-cells (red) as defined by CD19 expression only.

**FIGURE 3:**
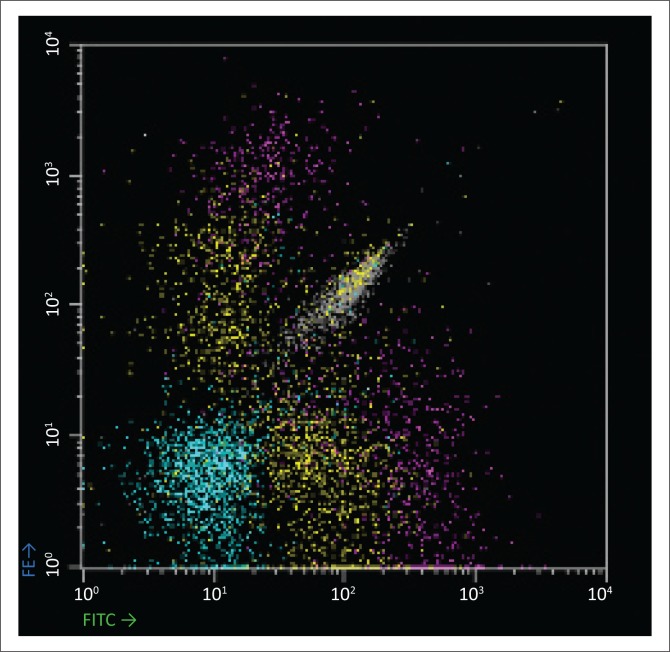
Immunophenotypic analysis of the peripheral blood performed on a dual laser FACSCalibur equipped with CellQuest Pro and PAINT-A-Gate Pro software. Kappa is the monoclonal antibody on FITC, and lambda is the monoclonal antibody on PE. There is a population of polyclonal plasma cells (yellow population), polyclonal B-cells (violet population) and T-cells (cyan).

Baseline laboratory investigations were performed (Table 1). The full blood count (FBC) revealed a leucocytosis with a lymphocytosis and eosinophilia. The peripheral blood smear (PBS) demonstrated 31% atypical lymphocytes and plasmacytoid lymphocytes (Figure 1). A lymphoproliferative neoplasm associated with HIV infection was considered. Flow cytometry of the peripheral blood was performed. Immunophenotypic analysis revealed a population of 20% – 25% reactive plasma cells with a range of CD138 (dim to +++) expression (Figure 2) and no light chain restriction (Figure 3). In addition, there were ~26% – 28% reactive T-cells and ~8% polyclonal B-cells (Figure 3). Polymerase chain reaction analysis for the immunoglobulin heavy-chain gene rearrangement studies was polyclonal. These findings demonstrate no evidence of a B-cell lymphoproliferative disorder.

**TABLE 1:** Baseline laboratory investigations.

Investigations	Result (reference range)
White blood cell count	41.1 × 10^9^/L (3.9–12.9)
Neutrophils	17.6 × 10^9^/L (1.6–8.3)
Lymphocytes	6.3 × 10^9^ /L (1.4–4.5)
Eosinophils	3.1 × 10^9^/L (0.0–0.4)
Alanine transaminase (ALT)	66 U/L (7–35)
Aspartate transaminase (AST)	41 U/L (13–35)
Alkaline phosphatase (ALP)	116 U/L (42–98)
Gamma-glutamyl transferase (GGT)	145 U/L (< 40)
Urea	4.3 mmol/L (2.1–7.1)
Creatinine	75 μmol/L (49–90)
C-reactive protein (CRP)	30 mg/L (< 10)
Erythrocyte sedimentation rate (ESR)	45 mm/h (0–10)
Blood cultures	Negative
Hepatitis B and C serology	Negative

Further laboratory and radiologic investigations supported the diagnosis of DRESS Syndrome (Table 2).^1^ The chest X-ray revealed bilateral interstitial lung infiltrates in keeping with pneumonitis. The liver function tests (LFT) were abnormal (Table 1). Serology for hepatitis studies was negative. Additional viral studies and a skin biopsy were not performed, at the discretion of the treating physician.

**TABLE 2:** Clinical and laboratory presentation criteria.

Presentation criteria for DRESS diagnosis in this patient
Hospitalisation†
Reaction suspected to be drug related†
Acute rash†
Fever > 38 °C†,‡
Enlarged lymph nodes involving at least two sites†,‡
Involvement of at least one internal organ^a^ Liver abnormalities‡
Blood count abnormalities†,‡
Lymphocytosis above normal limits†,‡
Eosinophils above laboratory limits†,‡
Leucocytosis (> 11 × 10^9^/L)‡
Atypical lymphocytosis (> 5%)‡

DRESS, drug reaction with eosinophilia and systemic symptom.

† , Three out of four of the RegiSCAR criteria are required for the diagnosis of DRESS;

‡ , Seven out of ten of the Japanese groups’ criteria are required for the diagnosis of DRESS.

Sulfasalazine was immediately discontinued. Administration of promethazine, montelukast as well as intravenous and topical hydrocortisone led to a dramatic improvement. The clinical manifestations resolved and the patient was discharged. On outpatient follow-up, laboratory investigations, namely FBC, PBS and LFT, had returned to baseline.

## Ethical consideration

Ethics was indeed obtained from the University of the Witwatersrand’s ethics committee. This was a retrospective case report and consent was therefore not a requirement.

## Discussion

Drug reaction with eosinophilia and systemic symptoms, also known as drug-induced hypersensitivity syndrome, is an adverse drug reaction commonly associated with numerous drug classes, including anticonvulsants, sulphonamides, antidepressants, anti-inflammatory drugs, antibiotics, angiotensin-converting enzymes and beta blockers.^1^ The last drug prescribed may not always be the offending drug, as the hypersensitivity reaction has a delayed onset of up to three months. This is often difficult in patients infected with HIV on multiple drugs. Further, these patients are at high risk of developing concomitant opportunistic infections, and DRESS often mimics non-specific infections. It is thus always important to keep drug history in mind when evaluating patients infected with HIV.

Drug hypersensitivity reactions are common in patients infected with HIV. Drugs prescribed for the treatment of HIV, namely reverse transcriptase inhibitors and protease inhibitors, as well as drugs for the prevention of opportunistic illnesses, namely sulphonamides, dapsone and anti-tuberculosis drugs, predispose these patients to drug reactions.^2,3^ In addition, there are numerous reports of adverse drug hypersensitivity reactions in this patient population to other drug classes. Drug reaction with eosinophilia and systemic symptoms associated with sulfasalazine in a patient infected with HIV to our knowledge has not been previously reported. Sulfasalazine is a modified sulphonamide composed of sulfapyridine covalently linked to 5-aminosalacyclic acid for the treatment of ulcerative colitis and RA.

The pathogenesis of DRESS is not fully understood. In most drugs implicated in DRESS, an association with lymphocyte activation, genetic drug metabolising enzyme defects or eosinophilic tissue infiltration has been described.^4,5^ A deficiency of detoxifying enzymes results in the accumulation of drug metabolites.^4^ Certain human leucocyte antigen (HLA) alleles may predispose to drug reactions because of naïve T-cells recognising a HLA–hapten complex on an antigen-presenting cell with a subsequent hyperactive immune response.^2^ More recently, reactivation of human herpes virus family has been associated with the diagnosis of a more severe form of DRESS, namely drug-induced hypersensitivity syndrome.^6^

The diagnosis of DRESS is based on laboratory and clinical criteria. However, there is a significant overlap with other reactive and malignant disorders. Classical haematologic abnormalities include a leucocytosis, eosinophilia and atypical lymphocytosis.^7^ Reactive plasma cells in the peripheral blood have been described in single case reports; however, a marked plasmacytosis as described in this case is rare. A marked peripheral blood plasmacytosis is characteristically associated with haematological malignancies such as plasma cell neoplasms and terminally differentiated mature lymphoproliferative B-cell neoplasms. In the setting of HIV, plasmacytoid and atypical lymphocytes are frequently described; however, plasma cells on PBS are also a rare finding.^8^ Infections with other viruses such as Epstein-Barr virus, parvovirus, hepatitis or cytomegalovirus should also be excluded.

The most common clinical findings are a mucocutaneous rash with or without systemic symptoms which include fever, lymphadenopathy and multi-organ dysfunction. As described in this patient, the most commonly involved internal organ is the liver.^5^ Other organs such as the kidney, lungs, heart or central nervous system are rarely involved.^3^ This is associated with a significant morbidity and a mortality of up to 10%, if the precipitating drug is not immediately discontinued.^3^ Supportive treatment, which depends on the severity of the clinical signs, is the mainstay of treatment. Isolated mucocutaneous involvement usually responds to topical corticosteroids, whereas systemic corticosteroids may be indicated in the presence of severe mucocutaneous lesions or other organ involvement. The role for systemic corticosteroids in patients with liver impairment, however, is not well established.^4^ Counselling of family members for a possible genetic susceptibility to DRESS is also advised.

In conclusion, this case report describes a drug reaction to sulfasalazine which presented atypically with a marked peripheral blood plasmacytosis mimicking a lymphoproliferative neoplasm. The diagnosis of DRESS in HIV-positive patients on multiple drugs with concomitant disorders presents a diagnostic dilemma. It is important to consider drug reactions when evaluating patients infected with HIV.

## References

[1] Kardaun SH, Sidoroff A, Valeyrie-Allanore L, et al Variability in the clinical pattern of cutaneous side-effects of drugs with systemic symptoms: Does a DRESS syndrome really exist? Br J Dermatol. 2007;156(3):609–11. https://doi.org/10.1111/j.1365-2133.2006.07704.x 17300272 10.1111/j.1365-2133.2006.07704.x

[2] Criado PR, Criado RFJ, Avancini J, Santi CG Drug reaction with Eosinophilia and Systemic Symptoms (DRESS)/Drug-induced Hypersensitivity Syndrome (DIHS): A review of current concepts. An Bras Dermatol. 2012;87(3):435–449. https://doi.org/10.1590/S0365-05962012000300013 22714760 10.1590/s0365-05962012000300013

[3] Cacoub P, Musette P, Descamps V, et al The DRESS syndrome: A literature review. Am J Med. 2011;124:588–597. https://doi.org/10.1016/j.amjmed.2011.01.017 21592453 10.1016/j.amjmed.2011.01.017

[4] Choudry S, McLeod M, Romanelli P Drug Reaction with Eosinophilia and Systemic Symptoms (DRESS) syndrome. J Clin Aesthet Dermatol. 2013;6(6):31–37. 23882307 PMC3718748

[5] Kano Y, Shiohara T Sequential reactivation of herpesvirus in drug-induced hypersensitivity syndrome. Acta Derm Venereol. 2004;84:484–485. https://doi.org/10.1080/00015550410016976 15844647

[6] Yoshikawa T, Fujita A, Yagami A, et al Human herpesvirus 6 reactivation and inflammatory cytokine production in patients with drug induced hypersensitivity syndrome. J Clin Virol. 2006;37 Suppl 1:S92–S96. https://doi.org/10.1016/S1386-6532(06)70019-1 17276377 10.1016/S1386-6532(06)70019-1

[7] Gentile I, Talamo M, Borgia G Is the drug-induced hypersensitivity syndrome (DIHS) due to herpesvirus 6 infection or to allergy-mediated viral reactivation? Report of a case and literature review. BMC Infect Dis. 2010;10:49 https://doi.org/10.1186/1471-2334-10-49 20205923 10.1186/1471-2334-10-49 PMC2845584

[8] Opie J Haematological complications of HIV infection. S Afr Med J. 2012;102(6):465–468. https://doi.org/10.7196/SAMJ.5595 22668938 10.7196/samj.5595

